# Baseline data report of the China Dialysis Outcomes and Practice Patterns Study (DOPPS)

**DOI:** 10.1038/s41598-020-79531-4

**Published:** 2021-01-13

**Authors:** Xinju Zhao, Qingyu Niu, Liangying Gan, Fan Fan Hou, Xinling Liang, Zhaohui Ni, Yuqing Chen, Junhui Zhao, Brian Bieber, Bruce Robinson, Xiaonong Chen, Li Zuo

**Affiliations:** 1 Department of Nephrology, Peking University People’s Hospital, Unit 10C in Ward Building, 11 Xizhimennan Street, Xicheng District, Beijing, 100044 China; 2 Division of Nephrology, Nanfang Hospital, National Clinical Research Center for Kidney Disease, State Key Laboratory of Organ Failure Research, Southern Medical University, 1838 North Guangzhou Avenue, Guangzhou, China; 3 Division of Nephrology, Guangdong Provincial People’s Hospital, Guangdong Academy of Medical Sciences, Guangzhou, China; 4 Renal Division, Renji Hospital, Shanghai Jiaotong University School of Medicine, Shanghai, China; 5 Renal Division, Peking University First Hospital, Beijing, China; 6 Arbor Research Collaborative for Health, Ann Arbor, MI USA; 7 Department of Nephrology, Ruijin Hospital, Shanghai Jiaotong University School of Medicine, Shanghai, China

**Keywords:** Haemodialysis, Outcomes research

## Abstract

The number of patients on hemodialysis (HD) is rapidly increasing in China. As an Asian country with a large number of HD patients, understanding the status of Chinese HD patients has a special significance. We reported here the baseline data for China Dialysis Outcomes and Practice Pattern Study Phase 5 (DOPPS5). The DOPPS is an international prospective, observational cohort study. Patients were restricted to the initial sample of patients who participated in China DOPPS5. We summarized the baseline demographic and clinical data of patients. Results were weighted by facility sampling fraction. 1186 patients were initial patients in China DOPPS5. The mean age was 58.7 ± 3.5 years, with 54.6% males. The median dialysis vintage was 3.4 (1.5, 6.3) years. The main assigned primary end-stage kidney disease (ESKD) causes was chronic glomerulonephritis (45.9%), followed by diabetes (19.9%). 17.6% patients had hepatitis B infection, and 10.0% patients had hepatitis C infection. 25.9% patients had a single-pooled Kt/V < 1.2. 86.6% patients had albumin > 3.5 g/dl. 18.8% patients had hemoglobin < 9 g/dl. 66.5% patients had serum calcium in target range (8.4–10.2 mg/dl), 41.5% patients had serum phosphate in target range (3.5–5.5 mg/dl) and 51.2% patients maintained PTH in 150–600 pg/dl. 88.2% patients used fistula as their vascular access. Meanwhile, there were differences in the demographic, clinical, laboratory, and treatment characteristics among the three cities participated in China DOPPS. We observed a relatively higher albumin level and a higher rate of fistula usage in our patients. But it remains a major challenge to us on the management of CKD-MBD and anemia. This study did not include patients in small cities and remote areas, where the situation of HD patients might be worse than reported.

## Introduction

The population of patients initiating and living with hemodialysis (HD) is increasing each year in China. It was reported that prevalent HD patients in China increased from 174.1 PMP in 2011 to 379.1 PMP in 2017^1^. This number will keep growing in the coming years. Due to the large population of end‐stage kidney disease (ESKD) patients in China, it is crucial to explore and understand more about this country’s dialysis practices. As a populous country in Asia, China is very different from developed countries and western countries in terms of race, economy, medical insurance policies, and medical practice patterns, such as more patients on twice-weekly dialysis treatments^2^, different available medication, and so on. However, data on HD patients’ characteristics and practice patterns from a large sample size remain sparse.

China has a vast territory and unbalanced economic development. Previous studies have found that the patients’ characteristics and dialysis practice patterns in different regions within China were not the same, but those research results were limited by the ununiformed research methods.

Dialysis Outcomes and Practice Patterns Study (DOPPS) is an international prospective cohort study of hemodialysis (HD) patients^3^. Previously, few studies reported comprehensive information about HD practices and patients’ situations at the national level in China. Exploring HD patients’ current treatment status in China is important for seeking optimal practices for Chinese patients and fostering the exchange of real-world data across different countries and regions. This study also enables a comparison between different cities.

This article aims to present the baseline data of China DOPPS5 and compare details among different cities involved in this study. By reporting national level data of hemodialysis practice pattern, more comprehensively epidemiological data of dialysis patients in China will be obtained. This will provide insight into opportunities to improve dialysis practices and patient outcomes.

## Methods

### Study design and subjects

Begun in 1996, the DOPPS is an international prospective cohort study of HD patients including many countries, and details were described in previous articles^3,4^. In China, the three largest cities (Beijing, Guangzhou, and Shanghai) were selected to participate in the pilot study and to provide representative data^5,6^. Beijing, Shanghai, and Guangzhou are the largest cities in northern, central, and southern China, respectively, and these cities can generalize the conditions in different region of China. Within each city, 15 study sites were randomly selected from a stratified list of all HD facilities treating more than 25 HD patients. Within each study site, detailed data were collected from a random sample of 20–40 subjects with an average of 30. Every year, patients departing the study were replaced from the pool of patients who joined the facility since the last sampling period to maintain the target number of patients per facility and a random sample within the facility.

Simple data for all census patients and detailed data for sample patients were collected. Inclusion criteria for participants were age ≥ 18 years old, treated at an in-center dialysis clinic and receiving chronic, maintenance HD (with regular dialysis more than 3 months). Exclusion criteria: patients were < 18 years old, treated with a home-based dialysis modality or receiving HD for acute kidney injury. Data collection was implemented mainly through questionnaires. In DOPPS5 (2012–2015), dialysis prescription, laboratory values, and medications were collected at baseline and yearly after that. There were no new data collection or interventions were planned.

In the current China DOPPS5 analysis, we mainly reported hemodialysis practice patterns of Chinese HD patients, and shown the baseline data including demographics, laboratory values and comorbidities. This information will provide insight into strategies for improving hemodialysis services elsewhere in China. So, we restricted to initial prevalent cross-section patients who were participated in this study at initial. We used this approach to exclude replacement patients as they were typically incident patients. Baseline hepatitis B infection was defined as an established diagnosis of hepatitis B infection or HBsAg-positive/HBcAb-positive with HBsAb-negative. Baseline hepatitis C infection was determined based on an established diagnosis of hepatitis C infection or hepatitis C antibody was positive.

The authors confirm that all the methods used in this study comply with the ethical standards of the relevant national and institutional committees on human experimentation and with the Helsinki Declaration. The study conforms to the *STROBE* guideline (see “Supplementary Data STROBE Checklist”). This study was approved by the Ethics Committee of Peking University People’s Hospital (ethical approval number: 2018PHB028-01). And all patients signed the written informed consent.

### Statistics

Continuous variables were expressed as mean ± standard deviation or median (25th, 75th), and categorical data were expressed as percentage. Results were weighted by facility sampling fraction to be more representative of the overall population as there was a large range in facility size. Differences in mean and median values between three cities were analyzed by using PROC SURVEYREG procedure. Categorical data between groups were compared by using chi-square test. As this article was mainly descriptive, we did not apply statistical methods to fill the missing data. All the statistical analyses were performed using SAS 9.4 (SAS Institute Inc., Cary, NC) and Microsoft Excel 2019 (Microsoft Corp., Redmond, WA).

### Ethics approval

The study was approved by the Ethics Committee of Peking University People’s Hospital (ethical approval number: 2018PHB028-01).

### Consent to participate

All patients signed the written informed consent.

## Results

Among 1427 sample patients, 1186 patients participated in this study initially, and 241 were replaced patients. The mean age of initial patients was 58.7 ± 3.5 years, and 54.6% were males. The primary cause of ESKD was chronic glomerulonephritis (CGN) (45.9%), followed by diabetic nephropathy (DN) (19.9%), hypertensive nephropathy (15.7%). The median dialysis vintage was 3.4 years, and patients undergoing dialysis for more than 5 years accounted for 33.9%.

391 initial patients participated in China DOPPS5 in Beijing, 371 in Guangzhou and 424 in Shanghai. The proportion of males (49.2%) in Beijing was lower than in other groups. The median dialysis vintage in Shanghai patients was the longest (4.1 (2.0, 7.9) years), followed by Beijing (3.6 (1.3, 7.1) years) and Guangzhou (2.4 (1.0, 4.0) years). 35.5% patients in Beijing and 40.5% in Guangzhou had a urine output of more than 200 ml per day, but this proportion was only 18.2% in Shanghai. The mean BMI in Beijing was higher than the other two groups. And the proportion of smokers was lowest in Shanghai (29.0%). Among our patients, 22.5% had coronary heart disease, 21.0% had congestive heart failure, and 19.5% had other cardiovascular diseases. The proportion of comorbidities in 3 cities were also different. A higher proportion of patients in Beijing combined with the cardiovascular and cerebrovascular disease and lung disease than patients in Guangzhou and Shanghai (Table 1). Dialysate electrolyte concentrations varied widely among patients in the three cities (Supplementary Table 1).

**Table 1:** Demographics and patient characteristics of the cohort study by region.

Variables	All	Region			P
		Beijing	Guangzhou	Shanghai	
Sample patients	1427	473	454	500	
Initial sample patients	1186	391	371	424	
**Demographics**					
Age [years (mean ± SD)]	58.7 ± 3.5	58.4 ± 3.2	58.8 ± 3.4	58.9 ± 3.7	0.8924
Males	54.6	49.2	58.9	55.2	0.0756
Vintage (years)	3.4 (1.5, 6.3)	3.6 (1.3, 7.1)	2.4 (1.0, 4.0)	4.1 (2.0, 7.9)	< 0.0001*
Urine output > 200 ml/day (%)	30.9	35.5	40.5	18.2	< 0.0001*
BMI (kg/m^2^)	21.8 ± 3.6	22.7 ± 3.5	21.5 ± 3.7	21.3 ± 3.4	< 0.0001*
Smokers	32.8	31.0	40.4	29.0	0.0046*
Assigned primary ESKD causes					–
CGN	45.9	36.7	39.5	56.2	
DN	19.9	21.8	27.4	13.6	
HT	15.7	17.4	16.5	14.0	
Others	18.5	24.1	16.6	16.2	
**Dialysis prescription**					
spKt/V	1.4 ± 0.3	1.5 ± 0.3	1.2 ± 0.3	1.4 ± 0.3	< 0.0001*
stdKt/V	2.3 ± 0.3	2.3 ± 0.3	2.1 ± 0.3	2.3 ± 0.3	< 0.0001*
Intradialytic weight loss (kg)	2.3 (1.7, 3.0)	2.5 (1.7, 3.2)	2.2 (1.6, 2.9)	2.3 (1.7, 3.0)	0.0159*
Dialysis frequency					0.0028*
2-times per week	20.2	16.7	27.0	17.9	
3-times per week	79.8	83.3	70.0	82.1	
HDF	9.8	6.9	6.2	13.9	< 0.0017*
Vascular access type					0.0001*
Fistula	88.2	87.2	81.9	93.1	
Catheter	11.8	12.8	18.1	6.9	
Blood flow rate (ml/min)	230 (210, 250)	250 (230, 260)	200 (220, 230)	230 (220, 250)	< 0.0001*
**Laboratory values**					
Hgb (g/dl)	10.7 (9.6, 11.9)	11.1(10.0, 12.1)	10.2 (8.9, 11.6)	10.7 (9.6, 119)	< .0001*
ALB (g/dl)	4.0 (3.7, 4.2)	4.0 (3.8, 4.3)	3.9 (3.6, 4.1)	4.0 (3.8, 4.3)	< 0.0001*
Ferritin (ng/ml)	314.0 (134.1, 580.0)	476.2 (201.4, 806.0)	368.9 (174.0 760.0)	243.0 (101.3, 420.1)	< 0.0001*
TSAT (%)	29.0 (21.5, 39.0)	26.4 (20.4, 34.7)	27.9 (20.0, 39.0)	30.1 (23.0, 42.5)	0.0120*
cCa (mg/dl)	9.0 (8.5, 9.7)	9.1 (8.5, 9.7)	8.9 (8.3, 9.6)	9.1 (8.6, 9.8)	0.0198*
P (mg/dl)	5.7 (4.6, 7.2)	5.3 (4.4, 6.8)	6.1 (4.9, 7.7)	5.6 (4.7, 7.2)	< 0.0001*
iPTH (pg/dl)	279.3 (139.7, 513.0)	241.0 (135.2, 407.0)	344.9 (186.2, 697.2)	281.3 (131.0, 513.0)	< 0.0001*
Hepatitis B infection (%)	17.6	12.8	12.3	24.2	< 0.0001*
Hepatitis C infection (%)	10.0	6.5	4.1	16.1	< 0.0001*
**Comorbidities (%)**					
Coronary heart disease	22.5	28.9	16.3	22.3	< 0.0012*
Congestive heart failure	21.0	25.8	23.6	16.2	< 0.0054*
Calciphylaxis	8.5	11.3	7.9	7.2	0.1837
Cancer (nonskin)	3.4	6.5	3.7	1.3	0.0004*
Other cardiovascular disease	19.5	29.1	9.2	20.0	< 0.0001*
Carpel tunnel syndrome	1.9	1.6	1.0	2.7	0.2746
Cerebrovascular disease	13.1	14.8	7.2	15.8	0.0017*
GI bleeding	2.1	1.4	2.4	2.4	0.6248
HIV/AIDS	0.1	0.4	0	0	–
Hypertension	87.3	85.3	83.2	91.2	0.0026*
Hyperlipidemia	35.4	41.8	30.9	34.2	0.0268*
Lung disease	4.2	8.8	3.5	1.6	< 0.0001
Neurologic disease	2.4	2.3	4.2	1.2	0.0712
Peripheral vascular disease	7.1	9.7	7.3	5.2	0.0457
Recurrent cellulitis	1.3	1.7	1.9	0.7	0.2990
Psychologic disorder	2.4	3.6	2.3	1.8	0.3707

*BMI* body mass index, *CGN* chronic glomerulonephritis, *DN* diabetic nephropathy, *HT* hypertensive nephropathy, *spKt/V* single-pooled Kt/V, *stdKt/V* standardized Kt/V, *HDF* hemodiafiltration, *Hgb* hemoglobin, *ALB* albumin, *TSAT* transferrin saturation, *cCa* corrected calcium, *P* phosphate, *iPTH* intact parathyroid hormone, *PCS* physical component summary, *MCS* mental component summary.

### Assigned primary ESKD causes

**Figure 1:**
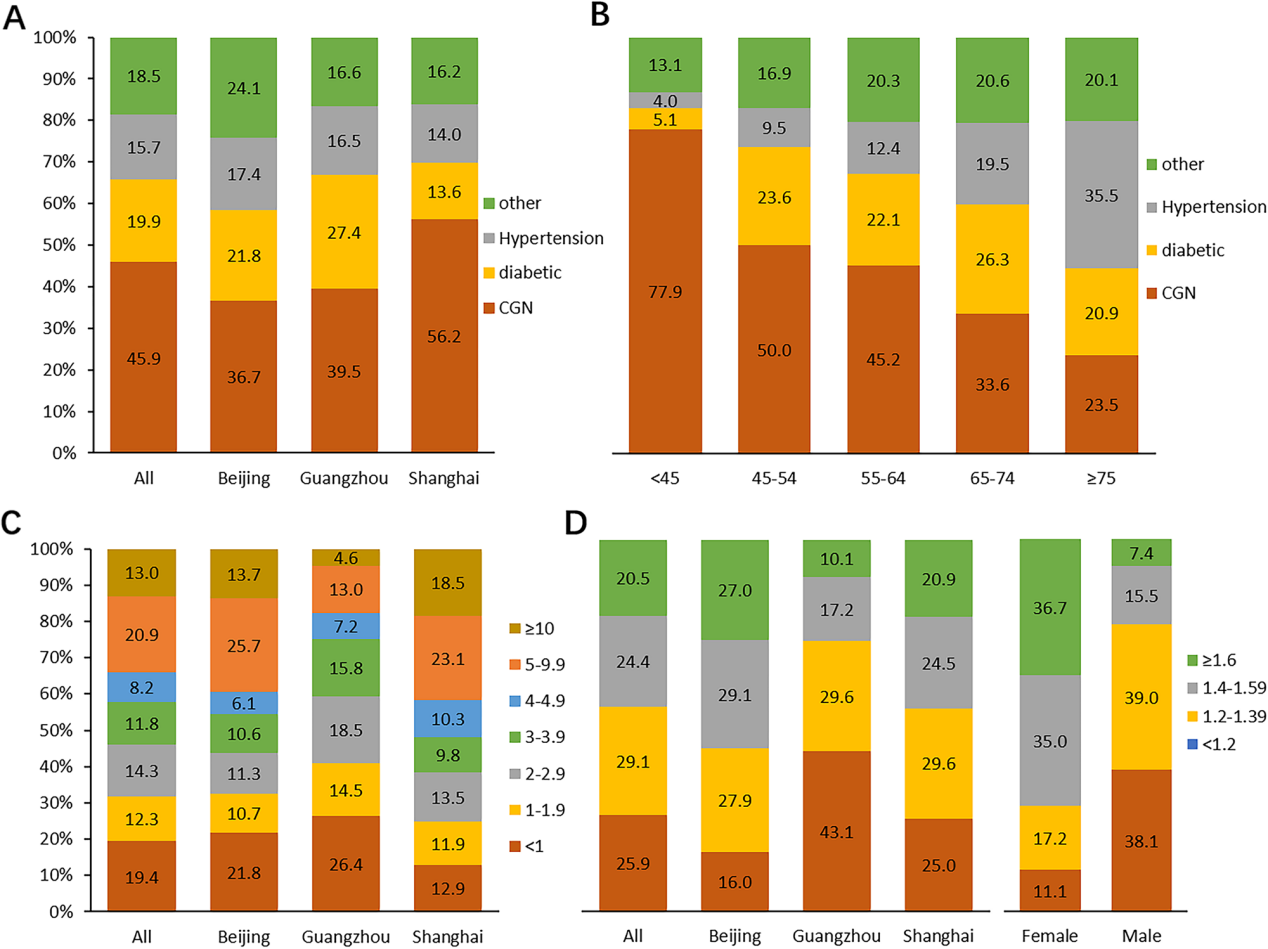
The distribution of the assigned primary ESKD causes, dialysis vintage and spKt/V. (**A**) The distribution of the assigned primary ESKD causes stratified by city group; (**B**) the distribution of the assigned primary ESKD causes stratified by age group; (**C**) the distribution of dialysis vintage stratified by city group; (**D**) the distribution of spKt/V stratified by city and gender groups.

For initial sample patients, assigned primary ESKD causes were predominantly by CGN (45.9%), followed by DN (19.9%), hypertensive nephropathy (15.7%) and others (18.5%). The proportion of primary ESKD causes varied from city to city. The proportion of CGN in Shanghai (56.2%) was significantly higher than that in Beijing and Guangzhou (36.7% and 39.5%, respectively), while the proportion of DN in Shanghai (13.6%) was significantly lower than the other two groups (Fig. 1A). Among patients < 45 years old, the proportion of patients with CGN as primary ESKD cause was extremely high (77.9%), while in older patients, the proportion of CGN was decreased and the proportions of DN and hypertensive nephropathy were increased (Fig. 1B).

### Dialysis vintage

There were 19.4% patients with dialysis vintage < 1 year, and 46.6% patients with dialysis vintage ranging from 1 to 5 years. Meanwhile, there were 33.9% patients with dialysis vintage > 5 years. Patients in Guangzhou had shorter dialysis vintage than Beijing and Shanghai patients. The proportion of patients with vintage in 5–10 years in Guangzhou (13.0%) was much lower than that in Beijing (25.7%) and Shanghai (23.1%) (Fig. 1C).

### Kt/V

Single-pooled (sp) Kt/V was missing in 33.6% patients. Among patients with Kt/V, there were 5.9% patients with spKt/V < 1.2, 29.1% patients in 1.2–1.39, 24.4% patients in 1.4–1.59, and 20.5% patients ≥ 1.6. Among patients in different cities, low spKt/V was more common in Guangzhou than that in Beijing and Shanghai. There were 43.1% patients in Guangzhou with spKt/V < 1.2 vs. 16.0% in Beijing and 25.0% in Shanghai. A significant higher proportion of male patients had low spKt/V (< 1.2) than females (38.1% vs. 11.1%) (Fig. 1D).

### Albumin

**Figure 2:**
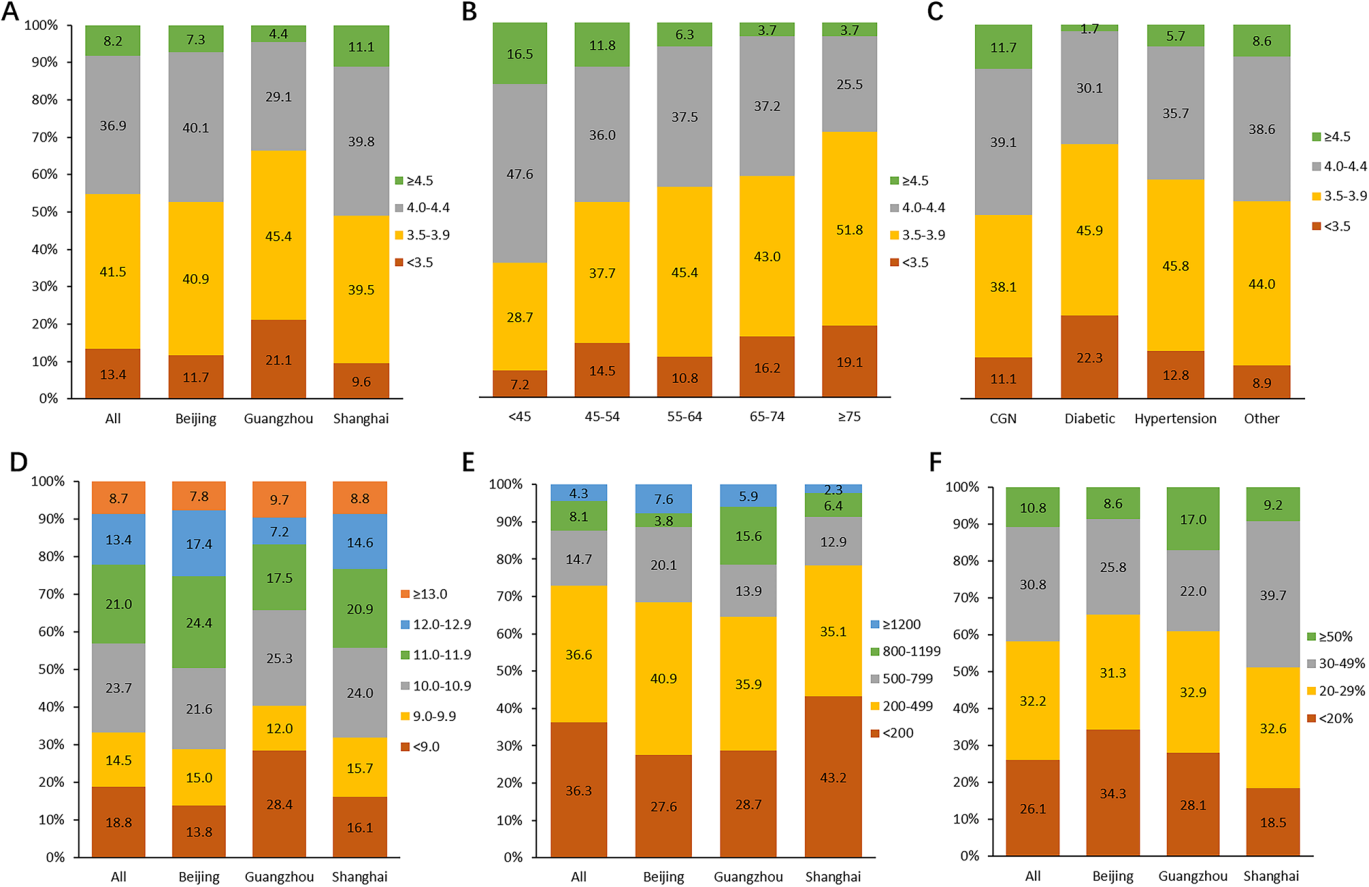
The distribution of ALB, Hgb, ferritin and TSAT. (**A**) The distribution of serum ALB level stratified by city group; (**B**) the distribution of serum ALB level stratified by age group; (**C**) the distribution of serum ALB level stratified by the primary ESKD causes group; (**D**) the distribution of serum Hgb level stratified by city group; (**E**) the distribution of serum ferritin level stratified by city group; (**F**) the distribution of TSAT stratified by city and gender group.

For serum albumin (ALB), there were 45.1% patients with ALB > 4.0 g/dl, 41.5% patients in 3.5–3.9 g/dl and 13.4% patients with ALB < 3.5 g/dl. There were more patients in Guangzhou with ALB < 3.5 g/dl than Beijing and Shanghai (Fig. 2A). Compared with younger patients, older patients had a lower ALB level (Fig. 2B). Meanwhile, patients with DN as the primary ESKD cause had a higher proportion of ALB < 3.5 g/dl than other groups (Fig. 2C).

### Anemia

18.8% patients in China DOPPS5 had hemoglobin (Hgb) < 9 g/dl and this percentage was relatively higher in Guangzhou (28.4%) comparing with Beijing (13.8%) and Shanghai (16.1%) (Fig. 2D). 36.3% patients had serum ferritin < 200 ng/ml and 27.1% patients had ferritin > 500 ng/ml. In Shanghai, a higher proportion of patients had ferritin < 200 ng/ml (43.2%) and a lower proportion of patients had ferritin > 500 ng/ml (21.6%) compared with Beijing and Guangzhou (Fig. 2E). Patients with transferrin saturation (TSAT) in 20–50% were accounted for 63.0%, and 26.1% patients with TSAT < 20%, 10.8% patients with TSAT > 50%. Patients in Shanghai had a higher proportion with TSAT in 20–50% (72.3%) than that in Beijing (57.1%) and Guangzhou (54.9%) (Fig. 2F).

### Chronic kidney disease-mineral and bone disorder (CKD-MBD) management

**Figure 3:**
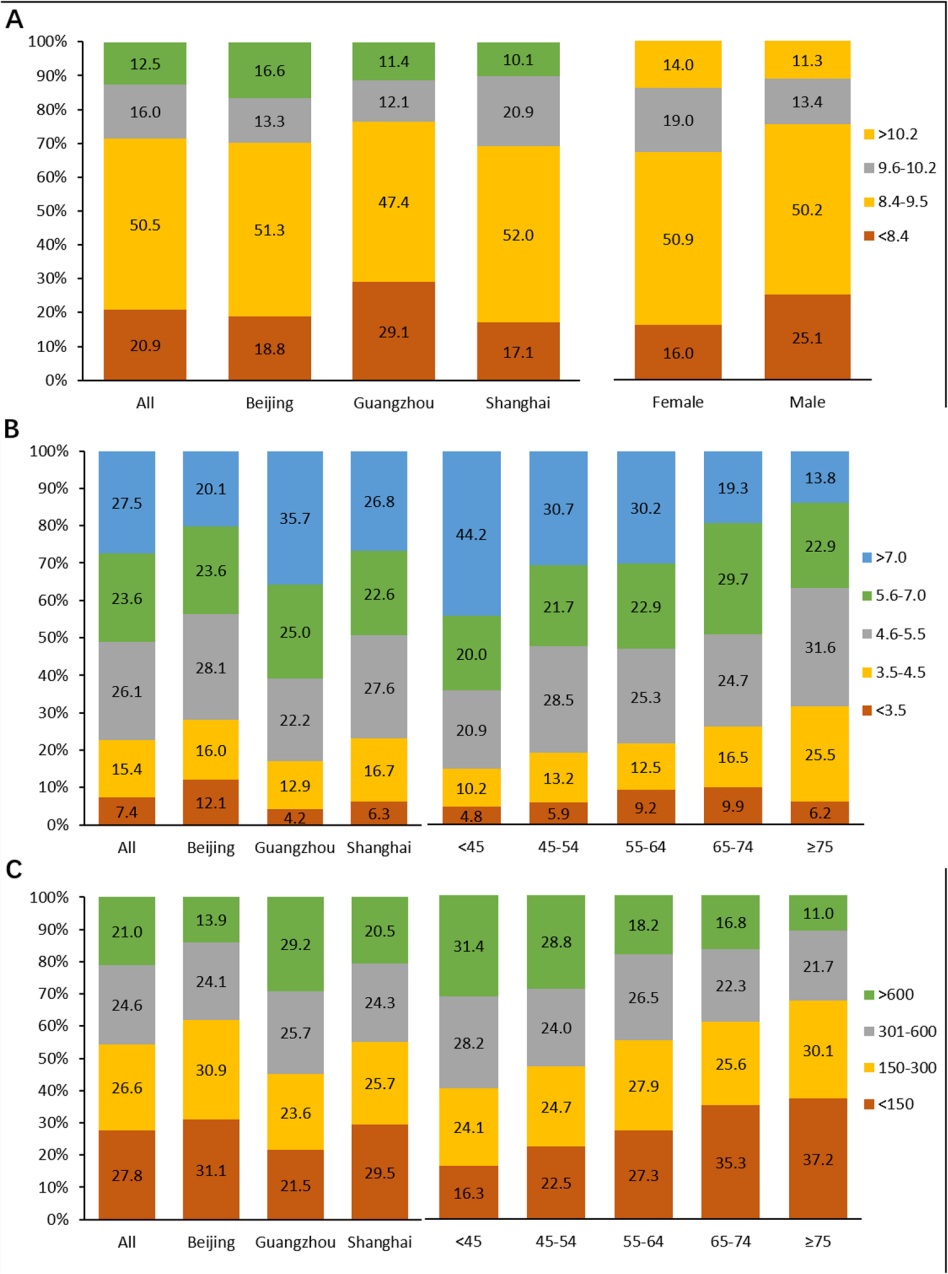
The distribution of bone mineral markers. (**A**) The distribution of serum cCa stratified by city and gender groups; (**B**) the distribution of serum P stratified by city and age groups; (**C**) the distribution of iPTH stratified by city and age groups.

66.5% patients had corrected calcium (cCa) within target range (8.4–10.2 mg/dl). More patients in Guangzhou (29.1%) had cCa < 8.4 mg/dl than Beijing and Shanghai (18.8% and 17.1%, respectively). Meanwhile, more male patients (25.1%) had cCa < 8.4 mg/dl compared with females (16.0%) (Fig. 3A). Serum phosphate (P) of our patients was not well controlled—only 41.5% patients with serum P level in the target range (3.5–5.5 mg/dl). There were 23.6% patients with serum P in 5.6–7.0 mg/dl and 27.5% patients with serum P > 7.0 mg/dl. Among cities, patients in Beijing (20.1%) had a much lower percentage of P > 7.0 mg/dl than Shanghai (26.8%) and Guangzhou (35.7%). Meanwhile, younger patients had a worse situation in serum P controlling. 44.2% of patients < 45 years old had serum P > 7.0 mg/dl (Fig. 3B). Only 51.2% patients had intact parathyroid hormone (iPTH) in the target range (150–600 pg/dl). More patients in Guangzhou had iPTH > 600 pg/dl (29.2%) compared with Beijing (13.9%) and Shanghai (20.5%). Older patients, especially patients ≥ 65 years old, had a higher percentage of PTH < 150 pg/dl and a lower percentage of PTH > 600 pg/dl than younger patients (Fig. 3C).

#### Hepatitis B and C infection

**Figure 4:**
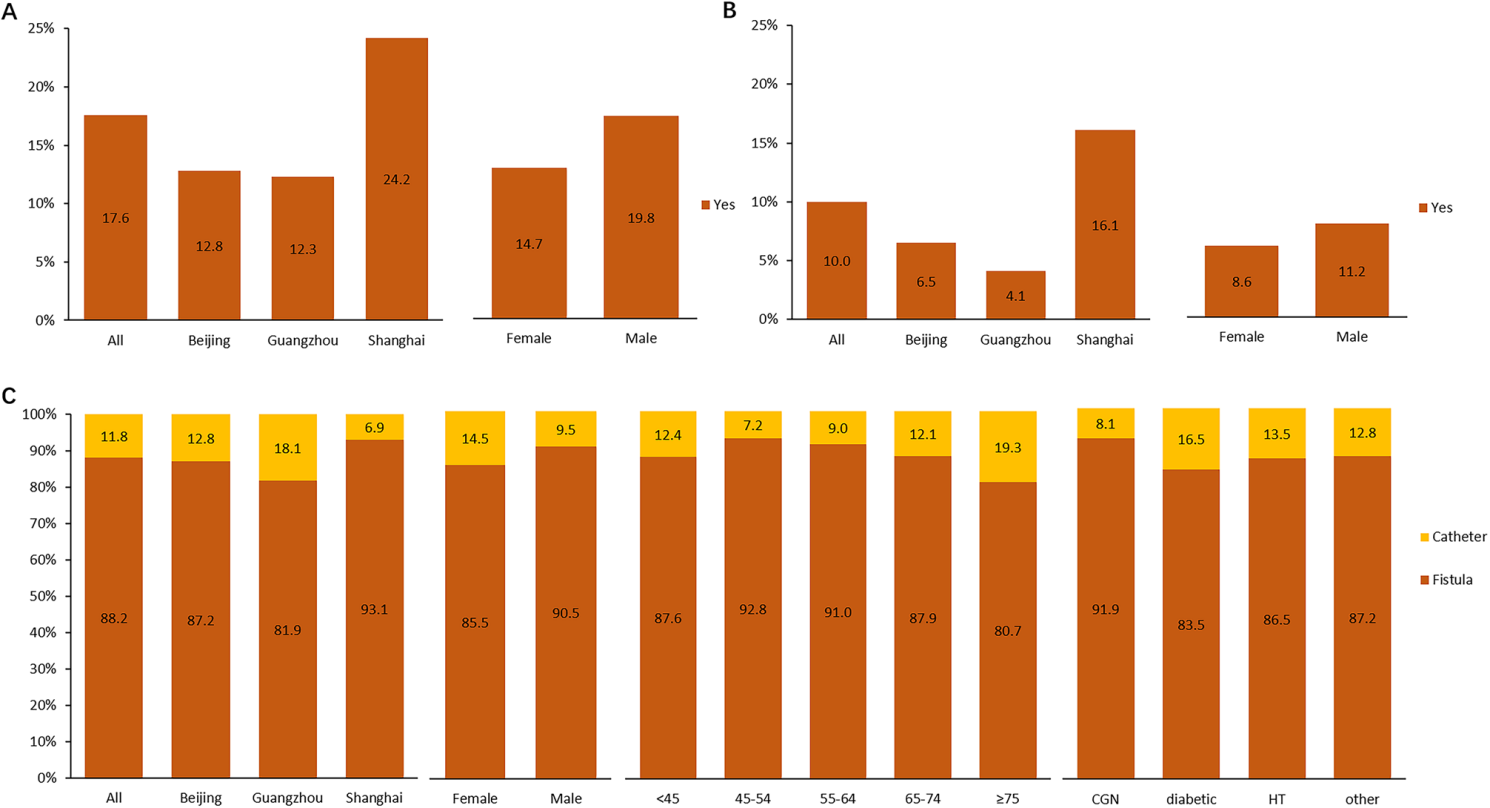
The distribution of hepatitis B infection, hepatitis C infection and vascular access. (**A**) The distribution of hepatitis B infection stratified by city and gender groups; (**B**) the distribution of hepatitis C infection stratified by city and gender groups; (**C**) the distribution of vascular access type stratified by city, gender, age and the primary ESKD causes groups.

The hepatitis B infection rate was 17.6% and the hepatitis C infection rate was 10.0% in our patients. Comparing with Beijing and Guangzhou, the hepatitis B infection rate was higher in Shanghai (24.2% v.s 12.8% and 12.3%). The hepatitis B infection rate in males (19.8%) was higher than females (14.7%) (Fig. 4A). Similarly, the hepatitis C infection rate in Shanghai (16.1%) was higher than the other two groups (6.5% in Beijing and 4.1% in Guangzhou) (Fig. 4B).

### Vascular access

Fistula was the predominant type of vascular access for China HD patients, accounted for 88.2%. This percentage was slightly higher among males. Patients in Shanghai had the highest rate of fistula use compared with other cities. In Shanghai, there were 93.1% patients were treated with fistula and only 6.9% were treated with catheter. In older patients, the rate of fistula use was lower than younger patients. Meanwhile, patients with diabetes tended to have a lower proportion of fistula use than other patients (Fig. 4C).

## Discussion

We reported the baseline demographic, clinical, laboratory and treatment characteristics data of HD patients in China DOPPS5 and compared the differences among cities (Beijing, Guangzhou and Shanghai). This randomly designed prospective cohort study, which performed in a representative study population, has provided some new insights in greater detail than currently available data. As the influences of different reimbursement policies and guidelines, substantial differences in dialysis treatment patterns and patients’ features existed by city. Therefore, there were some unique characteristics among HD patients in China.

While DN is the foremost cause of ESKD in developed countries^7^, CGN continues to be the most common cause of ESKD in China. The distribution of assigned primary ESRD causes was different: Shanghai has more patients with CGN and less with DN than Beijing and Guangzhou. Meanwhile, the proportion of CGN was much higher in younger patients.

Almost one-third of patients with a spKt/V < 1.2 in China DOPPS5, and patients in Guangzhou had a much higher proportion of Kt/V < 1.2 than Beijing and Shanghai. The standard-reaching rate of Kt/V was relatively low in Chinese HD patients. There were some possible reasons. First, a substantial number of HD patients with 2-times weekly dialysis in China as still have residual kidney function. Although their spKt/V did not meet the target, it may be acceptable as long as they meet a weekly standardized Kt/V target of 2.0 or above^8^. Meanwhile, more patients in Guangzhou were treated with 2-times weekly dialysis, which is consistent with the results of spKt/V. Second, the blood flow rate (BFR) of our patients was substantially lower than other countries in DOPPS. The most common BFR in our patients was 200 ml/min, compared with 300–500 ml/min in many other countries. A lower BFR was associated with lower Kt/V^9^.

Meanwhile, the prevalence of hepatitis B infection was relatively high in China^7^. Prevalence of hepatitis B and C both much higher in Shanghai than Beijing and Guangzhou. The much higher prevalence of hepatitis B and C in China indicated that we need further efforts to improve vaccination and infection control.

We also found that the achieved Ca, P and PTH levels were not optimal; even the management of CKD-MBD in China was based on the Kidney Disease: Improving Global Outcomes (KDIGO) guideline^10^. Among the three cities, Guangzhou has the worst results on CKD-MBD management. There were several reasons. First, due to economic and medical insurance issues, many patients are still undergoing 2-times weekly dialysis^11^. The relatively low frequency of dialysis may cause dialysis inadequate^12^. Second, a few years ago, some medicines with better curative effects, such as non-calcium phosphate binders, calcitriol and cinacalcet, had not entered the Chinese market or were not included in China's medical insurance coverage. This limited the application of optimal prescriptions to maintenance HD patients. With new drugs coming on the market, we assume that the CKD-MBD management in China will get better. Other probable reasons may include treatment time, dialysis schedule, patient compliance and so on.

Kidney anemia remains a noteworthy issue in Chinese HD patients as a substantial proportion of patients had low Hgb concentrations. A considerable proportion of our HD patients had Hgb < 9 g/dl^13,14^. Compared with DOPPS4 data in China, little improvement in anemia management was observed^6^. We also found that approximately one-third of patients had ferritin < 200 ng/ml and TSAT < 20%, which were also associated with higher odds of hypohemoglobinemia. There were other related reasons, such as the proportion of two-times weekly HD was higher than other DOPPS countries and the relatively low dose of erythropoietin stimulating agents^2^. We should focus more on HD patient’s comprehensive assessment and follow-up to facilitate early intervention and improve anemia management.

Nevertheless, we exhibited several positive factors in China. First of all, the rate of fistula use in China DOPPS5 was much higher than many DOPPS countries^15,16^. Many studies have shown that native fistula use was associated with lower mortality compared with the use of catheter^17–19^. There may be some reasons for the high use of fistula in China. First, our nephrologists adhered to the principle of ‘fistula first’. And, with the gradual improvement of CKD clinics, more patients have an opportunity to prepare vascular access for dialysis ahead of time. Then, most vascular access surgeries are performed by nephrologists, which saves the waiting time for patients to transfer to the department of surgery. Meanwhile, even if patients were initially treated with catheter, native fistula surgery will be performed at optimal timing.

Secondly, the serum ALB level of our patients was relatively high. Consistently, we have reported that serum ALB of Beijing HD patients was higher than many DOPPS countries in 2013^20^. Previous studies explored that low ALB was an independent predictor of mortality^21^. ALB was seen as a nutrition marker, and some researchers also considered it as a marker for inflammation because it can be negatively correlated with the systemic inflammation marker C-reactive protein^22^. However, the reason of the high ALB level of our patients was not clearly understood.

Despite the data collection on random samples of all HD facilities, our study has several limitations. First, there were several laboratory indices were not routinely tested in many facilities, such as TSAT, ferritin and Kt/V, so a higher proportion of these indices were missing. To reduce this limitation, in the future, dialysis facilities should comply with a standardized protocol to do laboratory tests regularly. Second, since the data were collected in three major cities of China and does not include patients receiving HD in smaller cities or rural areas, these results cannot be representative of the whole Chinese HD population. The situation of HD patients in whole China may be worse than what we reported.

The current situation of HD patients in China determines the direction we need to improve. We need to promote HD patients to undergo dialysis treatment no less than three times a week, improve dialysis adequacy, and increase HDF frequency. For the management of CKD-MBD, ensure that patients could receive adequate and beneficial treatment, such as increasing non-calcium phosphate binder use, strengthening diet management. Meanwhile, improve monitoring and calibration of erythropoietin stimulating agents to meet anemia targets. Through these measures, we will explore the optional dialysis practice patterns for Chinese HD patients.

## Conclusion

In conclusion, our analyses gave a detailed description of the demographic characteristics, treatment measures, practice patterns, and regional differences of Chinese HD patients. In terms of ALB level and fistula usage, our HD patients are doing well. However, we need to make more effort to improve the management of CKD-MBD and anemia, and lower the infection rate of hepatitis B and C. This study helps to better understand the current status of HD management in China. More researches designed to further understanding the status and practice patterns for Chinese HD patients are warranted.

## Supplementary Information

Supplementary Information.

Supplementary Table 1.

## Data Availability

The data used of this study are available from the corresponding author on reasonable request.

## Code availability

The code used of this study are available from the corresponding author on reasonable request.
