# Supplementary material for: Baseline data report of the China Dialysis Outcomes and Practice Patterns Study (DOPPS)
Source: Sci Rep. 2021 Jan 13;11:873. doi: 10.1038/s41598-020-79531-4 (PMC7806992; doi:10.1038/s41598-020-79531-4)
Supplement: Supplementary file 1 — Supplementary Information. [file 41598_2020_79531_MOESM1_ESM.docx]

STROBE Statement—checklist of items that should be included in reports of observational studies

|  | Item No. | Recommendation | Page  No. | Relevant text from manuscript |
| --- | --- | --- | --- | --- |
| **Title and abstract** | 1 | (*a*) Indicate the study’s design with a commonly used term in the title or the abstract | 3 | The DOPPS is an international prospective, observational cohort study. |
|  |  | (*b*) Provide in the abstract an informative and balanced summary of what was done and what was found | 3 | We observed a relatively high albumin level, high rate of fistula use in our patients. But it remains a major challenge to us on the management of CKD-MBD and anemia. This study did not include patients in small cities and in remote areas, where the situation of HD patients might be worse than reported. |
| Introduction | | | |  |
| Background/rationale | 2 | Explain the scientific background and rationale for the investigation being reported | 4 | Previous studies have found that the patients’ characteristics and dialysis practice patterns in different regions within China were not the same, but those research results were limited by the ununiformed research methods. |
| Objectives | 3 | State specific objectives, including any prespecified hypotheses | 5 | In this article, we aim to present the baseline data of China DOPPS5 and compare details among different cities involved in this study. |
| Methods | | | |  |
| Study design | 4 | Present key elements of study design early in the paper | 5 | Begun in 1996, the DOPPS is an international prospective cohort study of HD patients including many countries, and details were described in previous articles3,4. |
| Setting | 5 | Describe the setting, locations, and relevant dates, including periods of recruitment, exposure, follow-up, and data collection | 4-5 | In China, 3 largest cities (Beijing, Guangzhou, and Shanghai) were selected to participate in the pilot study and to provide representative data5,6. Data collection was implemented mainly through questionnaires. |
| Participants | 6 | (*a*) *Cohort study*—Give the eligibility criteria, and the sources and methods of selection of participants. Describe methods of follow-up  *Case-control study*—Give the eligibility criteria, and the sources and methods of case ascertainment and control selection. Give the rationale for the choice of cases and controls  *Cross-sectional study*—Give the eligibility criteria, and the sources and methods of selection of participants | 5 | Inclusion criteria for participants were age ≥ 18 years old, treated at an in-center dialysis clinic and receiving chronic, maintenance HD (with regular dialysis more than 3 months). Exclusion criteria: patients were <18 years old, treated with a home-based dialysis modality or receiving HD for acute kidney injury. |
|  |  | (*b*) *Cohort study*—For matched studies, give matching criteria and number of exposed and unexposed  *Case-control study*—For matched studies, give matching criteria and the number of controls per case | 5 | No match, just observation. There were no new data collection or interventions were planned. |
| Variables | 7 | Clearly define all outcomes, exposures, predictors, potential confounders, and effect modifiers. Give diagnostic criteria, if applicable | 5 | In this text, we mainly reported baseline data of China DOPPS5. |
| Data sources/ measurement | 8* | For each variable of interest, give sources of data and details of methods of assessment (measurement). Describe comparability of assessment methods if there is more than one group | *5* | The reported data including demographics, laboratory values and comorbidities. Baseline hepatitis B infection was defined as an established diagnosis of hepatitis B infection or HBsAg-positive/HBcAb-positive with HBsAb-negative. Baseline hepatitis C infection was determined based on an established diagnosis of hepatitis C infection or hepatitis C antibody was positive. |
| Bias | 9 | Describe any efforts to address potential sources of bias | 5 | Random. Within each city, 15 study sites were randomly selected from a stratified list of all HD facilities treating more than 25 HD patients. Within each study site, detailed data were collected from a random sample of 20-40 subjects with an average of 30. |
| Study size | 10 | Explain how the study size was arrived at | 5 | Within each study site, detailed patient data will be collected from a random sample of 20 to 40 subjects, yielding a total sample of approximately 1,400 hemodialysis patients. |

Continued on next page

| Quantitative variables | 11 | Explain how quantitative variables were handled in the analyses. If applicable, describe which groupings were chosen and why | 6 | Differences in mean and median values between 3 cities were analyzed by using PROC SURVEYREG procedure. |
| --- | --- | --- | --- | --- |
| Statistical methods | 12 | (*a*) Describe all statistical methods, including those used to control for confounding | 6 | Results were weighted by facility sampling fraction to be more representative of the overall population as there was a large range in facility size. |
|  |  | (*b*) Describe any methods used to examine subgroups and interactions |  | No subgroups. |
|  |  | (*c*) Explain how missing data were addressed | 6 | As this article was mainly descriptive, we did not apply statistical methods to fill the missing data. |
|  |  | (*d*) *Cohort study*—If applicable, explain how loss to follow-up was addressed  *Case-control study*—If applicable, explain how matching of cases and controls was addressed  *Cross-sectional study*—If applicable, describe analytical methods taking account of sampling strategy |  | Not applicable |
|  |  | (*e*) Describe any sensitivity analyses |  | Not applicable |
| Results | | | | |
| Participants | 13* | (a) Report numbers of individuals at each stage of study—eg numbers potentially eligible, examined for eligibility, confirmed eligible, included in the study, completing follow-up, and analysed | 7 | Among 1427 sample patients, 1186 patients were participated in this study at initial, and 241 were replacement patients. |
|  |  | (b) Give reasons for non-participation at each stage | 7 | Among 1427 sample patients, 1186 patients were participated in this study at initial, and 241 were replacement patients. |
|  |  | (c) Consider use of a flow diagram |  | No need |
| Descriptive data | 14* | (a) Give characteristics of study participants (eg demographic, clinical, social) and information on exposures and potential confounders | 7 | The mean age of initial patients was 58.7±3.5 years, and 54.6% were males. |
|  |  | (b) Indicate number of participants with missing data for each variable of interest | 8 | For initial sample patients, assigned primary ESKD causes were predominantly by CGN (45.9%), followed by DN (19.9%), hypertensive nephropathy (15.7%) and others (18.5%). (others include missing) |
|  |  | (c) *Cohort study*—Summarise follow-up time (eg, average and total amount) | 5 | DOPPS5 (2012-2015) |
| Outcome data | 15* | *Cohort study*—Report numbers of outcome events or summary measures over time |  | Not applicable. |
|  |  | *Case-control study—*Report numbers in each exposure category, or summary measures of exposure |  |  |
|  |  | *Cross-sectional study—*Report numbers of outcome events or summary measures |  |  |
| Main results | 16 | (*a*) Give unadjusted estimates and, if applicable, confounder-adjusted estimates and their precision (eg, 95% confidence interval). Make clear which confounders were adjusted for and why they were included |  | Not applicable. |
|  |  | (*b*) Report category boundaries when continuous variables were categorized | 10-11 | Categorized range was reported for each variable |
|  |  | (*c*) If relevant, consider translating estimates of relative risk into absolute risk for a meaningful time period |  | Not applicable. |

Continued on next page

| Other analyses | 17 | Report other analyses done—eg analyses of subgroups and interactions, and sensitivity analyses | 11 | No such analysis |
| --- | --- | --- | --- | --- |
| Discussion | | | | |
| Key results | 18 | Summarise key results with reference to study objectives | 11 | We reported the baseline demographic, clinical, laboratory and treatment characteristics data of HD patients in China DOPPS5. This random designed prospective cohort study, which performed in a representative study population, has provided some new insights in greater details than currently available data. Three major Chinese cities – Beijing, Guangzhou and Shanghai participated in this study. As the influences of different reimbursement policies and guidelines, substantial differences in dialysis treatment patterns and patients’ features existed by city and country. Therefore, there were some unique characteristics among HD patients in China compared with other DOPPS countries. |
| Limitations | 19 | Discuss limitations of the study, taking into account sources of potential bias or imprecision. Discuss both direction and magnitude of any potential bias | 14 | Despite the data collection on random samples of all HD facilities, our study has several limitations. First, there were several laboratory indices were not routinely tested in many facilities, such as TSAT, ferritin and Kt/V, so these indices were missing a higher proportion than other indices. To reduce this limitation, in the future, dialysis facilities should be more standardized and regular to do laboratory tests. Second, since the data were collected in three major cities of China and does not include patients receiving HD in smaller cities or rural areas, these results cannot be representative of the whole Chinese HD population. The situation of HD patients in whole China may be worse than what we reported. |
| Interpretation | 20 | Give a cautious overall interpretation of results considering objectives, limitations, multiplicity of analyses, results from similar studies, and other relevant evidence | 14 | Despite the data collection on random samples of all HD facilities, our study has several limitations. First, there were several laboratory indices were not routinely tested in many facilities, such as TSAT, ferritin and Kt/V, so these indices were missing a higher proportion than other indices. To reduce this limitation, in the future, dialysis facilities should be more standardized and regular to do laboratory tests. Second, since the data were collected in three major cities of China and does not include patients receiving HD in smaller cities or rural areas, these results cannot be representative of the whole Chinese HD population. The situation of HD patients in whole China may be worse than what we reported. |
| Generalisability | 21 | Discuss the generalisability (external validity) of the study results | 14 | Due to measurable differences in race, economy, medical insurance policies and dialysis facility practices, our situation was different from developed countries and western countries. The current situation of HD patients in China determines the direction we need to improve. Unless developed countries, we need to promote HD patients to undergo dialysis treatment no less than 3 times a week, improve dialysis adequacy, and increase the frequency of HDF. For the management of CKD-MBD, ensure that patients could receive an adequate and beneficial treatment, such as increasing the use of non-calcium phosphate binder, strengthening diet management. Meanwhile, improve monitoring and calibration of erythropoietin stimulating agents to meet anemia targets. Exploring the optional dialysis practice patterns for Chinese HD patients. |
| Other information | |  | | |
| Funding | 22 | Give the source of funding and the role of the funders for the present study and, if applicable, for the original study on which the present article is based | 15 | This article was supported by National Natural Science foundation of China, and the grant recipient is Li ZUO, grant number is 81870524. |

*Give information separately for cases and controls in case-control studies and, if applicable, for exposed and unexposed groups in cohort and cross-sectional studies.

**Note:** An Explanation and Elaboration article discusses each checklist item and gives methodological background and published examples of transparent reporting. The STROBE checklist is best used in conjunction with this article (freely available on the Web sites of PLoS Medicine at http://www.plosmedicine.org/, Annals of Internal Medicine at http://www.annals.org/, and Epidemiology at http://www.epidem.com/). Information on the STROBE Initiative is available at www.strobe-statement.org.
