# Supplementary material for: Baseline data report of the China Dialysis Outcomes and Practice Patterns Study (DOPPS)
Source: Sci Rep. 2021 Jan 13;11:873. doi: 10.1038/s41598-020-79531-4 (PMC7806992; doi:10.1038/s41598-020-79531-4)
Supplement: Supplementary file 2 — Supplementary Table 1. [file 41598_2020_79531_MOESM2_ESM.docx]

**Supplementary data**

**Baseline Data Report of the China Dialysis Outcomes and Practice Patterns Study (DOPPS)**

Xinju Zhao^1, a^, Qingyu Niu^1, a^, Liangying Gan^1^, Fan Fan Hou^2^, Xinling Liang^3^, Zhaohui Ni^4^, Yuqing Chen^5^, Junhui Zhao^6^, Brian Bieber^6^, Bruce Robinson^6^, Xiaonong Chen^7*^ Li Zuo ^1*^

1. Department of Nephrology, Peking University People’s Hospital, Beijing, China
2. Division of Nephrology, Nanfang Hospital, Southern Medical University, National Clinical Research Center for Kidney Disease, State Key Laboratory of Organ Failure Research, 1838 North Guangzhou Avenue, Guangzhou, China.
3. Division of Nephrology, Guangdong Provincial People's Hospital, Guangdong Academy of Medical Sciences, China
4. Renal Division, Renji Hospital, Shanghai Jiaotong University School of Medicine, Shanghai, China
5. Renal Division, Peking University First Hospital, Beijing, China
6. Arbor Research Collaborative for Health, Ann Arbor, Michigan, United States of America
7. Department of Nephrology, Ruijin Hospital, Shanghai Jiaotong University School of Medicine,

Shanghai, China

a, Xinju Zhao and Qingyu NIU contributed equally to this work.

***Corresponding author:**

Li Zuo, Department of Nephrology, Peking University People’s Hospital, Unit 10C in Ward Building; 11 Xizhimennan Street, Xicheng District; Beijing, 100044; China. Tel :+86（10）88324008; Fax: :+86（10）88325018; Email: [zuoli@bjmu.edu.com](mailto:zuoli@bjmu.edu.com)

Xiaonong Chen, Department of Nephrology, Ruijin Hospital, Shanghai Jiaotong University School of Medicine, Shanghai, China. Email: cxn10419@rjh.com.cn

**Running head：Data report of China DOPPS5**

Supplementary table 1. Demographics and Patient Characteristics of the Study Cohort by region

| Variables | all | Region | | | P |
| --- | --- | --- | --- | --- | --- |
|  |  | Beijing | Guangzhou | Shanghai |  |
| **Dialysis prescription** |  |  |  |  |  |
| Dialysate Ca (mEq/l)  2.5  3.0  3.5 | 287(20.5)  1020 (71.5)  87 (6.2) | 121 (25.7)  345 (73.4)  2 (0.43) | 114 (26.0)  269 (61.3)  54 (12.3) | 52 (11.0)  406 (82.4)  31 (6.3) | <0.0001* |
| Dialysate K (mmol/l)  2.0  2.5  3.0 | 994 (71.2)  293 (21.0)  67 (4.8) | 196 (41.5)  242 (51.3)  32 (6.8) | 383 (86.5)  51 (11.5)  0 (0) | 415 (86.3)  0 (0)  35 (7.3) | <0.0001* |
| Dialysate Na (mmol/l)  <138  138  139-140  >140 | 172 (12.1)  608 (42.6)  568 (39.8)  79 (5.5) | 45 (9.5)  257 (54.3)  155 (32.8)  16 (3.4) | 75 (16.5)  84 (18.5)  248 (54.6)  47 (10.4) | 52 (10.4)  267 (53.4)  165 (33.0)  16 (3.2) | <0.0001* |

Notes: GI, gastrointestinal; SBP, systolic blood pressure DBP, diastolic blood pressure.
